# Oat Milk Tea Model System: Exploring the Stability of Milk Tea and the Bioaccessibility of Green Tea Polyphenols

**DOI:** 10.3390/foods12071402

**Published:** 2023-03-26

**Authors:** Sirui Qin, Ruyi Li, Mianhong Chen, Fanke Zeng, Yaping Dai, Guang Wu, Wei Zhou, Jihua Li

**Affiliations:** 1 Key Laboratory of Tropical Crop Products Processing of Ministry of Agriculture and Rural Affairs, Agricultural Products Processing Research Institute, Chinese Academy of Tropical Agricultural Sciences, Zhanjiang 524001, China; 2 College of Food Science & Technology, Huazhong Agricultural University, Wuhan 430070, China

**Keywords:** oat milk, green tea polyphenols, food matrices, in vitro digestion, bioaccessibility

## Abstract

Oat milk has become preferential because of its low calorie nature and high dietary fiber content, but its ability to “curdle” when mixed with tea can affect the consumer acceptability for oat milk tea. In this study, a model system for oat milk tea was made by combining oat milk and green tea extract to evaluate the impacts of the oat milk matrix and green tea extract concentration on the stability and polyphenol bioaccessibility. The stability analysis results showed that adding green tea extract to oat milk influenced the stability of the oat milk tea model systems. In contrast, the 3.0% fat oat milk tea model system exhibited a higher stability than the 1.5% fat oat milk tea model system. In simulated gastrointestinal digestive experiments, tea polyphenols in the oat milk tea model systems were relatively stable in oral and stomach digestive stages, while they clearly degraded in the small intestine digestive stage. Furthermore, the bioaccessibility of tea polyphenols was higher for the 3.0% fat oat milk tea model system than for the 1.5% fat oat milk tea model system, especially at low concentrations of green tea extracts (0.05%~0.25%). These results may provide a theoretical reference and data for the formulation of oat milk tea and the bioaccessibility of tea polyphenols in food matrices.

## 1. Introduction

Oats are universally acknowledged as a healthy foodstuff because of their nutritional properties, such as high dietary fiber, well-balanced proteins, and bioactive polyphenols [1]. In particular, oat β-glucan, as a soluble dietary fiber, is closely associated with reducing serum cholesterol and the risk of cardiovascular diseases [2]. Therefore, the consumption of oat-based products with high dietary fiber can achieve the same health benefits. Oat-based products commonly consumed in the diet are oatmeal, oat flours, oat flakes, and oat milk [3]. Among these oat-based products, oat milk has become one of the most popular owing to its delicious taste, good flavor, and high consumer acceptability [4]. It is produced using water and oat flour through the following steps: grinding, enzymatic hydrolysis, filtration, thermal treatment, and homogenization [4]. Many customers have switched to oat milk instead of bovine milk in their diet, especially those with bovine milk protein allergy, those with lactose intolerance, and vegans [5]. Oat milk does not contain bovine milk protein or lactose, which is compatible with vegan principles. Therefore, oat milk can replace bovine milk and be mixed into tea broth to make oat milk tea [6].

Tea, particularly green tea, is rich in polyphenols (e.g., catechins, anthocyanins, and phenolic acid), which are related to health benefits (e.g., antioxidant and anti-inflammatory effects and the prevention of cardiovascular and degenerative diseases) [7,8]. Researchers have reported that consuming green tea and dairy products at the same time helps to preserve the integrity and antioxidant activity of polyphenols during digestion [9]. However, during the processing and storage of oat milk tea, tea polyphenols in green tea extract (GTE) can interact hydrophobically and/or can hydrogen bond with the food matrix (e.g., proteins and polysaccharides), causing aggregation of the food matrix [9,10,11]. Meanwhile, binding between polyphenols and the food matrix can change the bioaccessibility of polyphenols after ingestion [12,13]. The bioaccessibility of tea polyphenols is defined as the content of tea polyphenols released from the food matrix, solubilized in the small intestine, and accessible for absorption after gastrointestinal digestion. There are numerous factors that might affect the bioaccessibility of polyphenols; one of the most important is the type of food matrix [12,13]. At present, studying the bioaccessibility of dietary compounds (such as polyphenols) in vivo is very challenging. In addition, in vivo trials are expensive and take a long time. Thus, in vitro gastrointestinal digestion models have been established as quick and effective methods to analyze the bioaccessibility of dietary compounds [14]. As is customary, an in vitro gastrointestinal digestion model includes three stages of digestion, oral, gastric, and intestinal, which can determine the bioaccessibility of polyphenols quickly and accurately.

Oat milk has garnered increasing attention in recent years, but there is less information on the physicochemical behavior of oat-based products (such as oat milk tea) during in vitro gastrointestinal digestion. So far, many studies have focused on the optimization of oat milk production and on the composition and functional properties of polyphenols in tea. Only a few studies have reported the influence of the food matrix and fat content on the bioaccessibility of tea polyphenols in beverages [15]. The study of Lamothe et al. indicated that cheese decreased the bioaccessibility of tea polyphenols when cheese and tea were consumed together, whereas milk and yogurt had no significant influence on the bioaccessibility of tea polyphenols [9]. Therefore, selecting suitable food matrix and tea to consume together is a crucial step in effectively directing the manufacture of milk tea and improving the nutritional value of food.

Previous studies have reported that the food matrix may have a significant effect on the release, transformation, and absorption of polyphenols [9]. Therefore, in this study, two types of oat milk with various fat levels (1.5% and 3%) were mixed with green tea extract in a certain ratio to investigate the stability of the oat milk tea model system. Additionally, the patterns of changes in the concentration and bioaccessibility of tea polyphenols (TPs) in green tea extract before and after digestion were studied using the in vitro gastrointestinal digestion model. This study will provide theoretical references and data support for the formulation of oat milk tea and the bioaccessibility of TPs in complex food systems.

## 2. Materials and Methods

### 2.1. Materials

Oat milk (Ollie Beverage Co., Ltd., Charlotte, NC, USA) and green tea were purchased from a local supermarket (Wal-Mart, Zhanjiang, China). The nutrition information per 100 mL of oat milk is shown in Table 1. Mucin, α-amylase, pepsin, and pancreatin were purchased from Sigma-Aldrich Co. (St Louis, MO, USA). Purified water was produced by Milliq Integral 3 system (Millipore, Darmstadt, Germany). Other chemical reagents were all of analytical purity.

### 2.2. Extraction of Green Tea

Twenty-five grams of green tea were extracted with distilled water at a 1:25 (*w*/*v*, g/mL) ratio for 30 min at 80 °C and then filtered under vacuum with Whatman filter paper. The process of extraction was repeated three times, and the filtrate was combined. The filtrate was concentrated using a rotary evaporator, and then the concentrated solution was freeze-dried. The green tea extract was stored at −20 °C for analysis.

### 2.3. Preparation of Oat Milk Tea Model System

The green tea extract was dissolved in distilled water to obtain a green tea solution. The oat milk tea was synthesized by mixing oat milk and various concentration of green tea solution with a volume ratio of 8:2 (*v*/*v*). The concentrations of green tea solution in the final oat milk tea were 0.05%, 0.1%, 0.25%, 0.5%, and 1%.

### 2.4. Determination of Total Phenolics

The total phenolics were determined by the Folin–Ciocalteu method [16]. The sample (0.1 mL) was mixed with 2.9 mL of distilled water and 0.25 mL of Folin–Ciocalteu reagent and left to stand for 5 min, and then 0.75 mL of 20% Na_2_CO_3_ solution was added to the mixture. After 30 min, the absorbance of the mixture was determined at 765 nm using a spectrophotometer (U-T6A, Yipu Instrument Manufacturing Co., Ltd., Shanghai, China). A standard curve of gallic acid (y = 0.0027x + 0.0667, R^2^ = 0.9997) was used to calculate the total phenolic concentration of the samples.

### 2.5. Determination of Polyphenol Binding Quantity

The polyphenol binding quantity was determined based on the method of Wu et al. [17]. The oat milk tea model system was centrifuged at 4 °C for 30 min at 12,000 rpm/min, and the supernatant was removed. The total phenolics in oat milk tea and phenolics in the supernatant were measured using the Folin–Ciocalteu method. Equation (1) was used to determine the quantity of polyphenols that bound to the oat milk:

(1) $$Q_{bound}\;\left(\mathrm{mg}\;\mathrm{GAE}/\mathrm{mL}\right)=\frac{M_{tp}-M_{sp}}{V}$$

where *M_tp_* is the mass of tea polyphenols in the oat milk tea, *M_sp_* is the mass of tea polyphenols in the supernatant, and *V* is the total volume of oat milk tea for analysis of the polyphenol binding quantity.

### 2.6. Stability Analysis of Oat Milk Tea Model System

The physical stability of the oat milk tea model system was analyzed using a LUMifuge Full-function stability analyzer (LUM, GMBH, Berlin, Germany) based on the method of Liu et al., with minor modifications [18]. The samples were loaded into rectangular PC cells (2 × 8 mm) and placed into the testing tank. The test parameters of samples were a centrifugation rate of 2000 rpm/min, a centrifugation temperature of 25 °C, a scan interval of 10 s, and a scan number of 300.

### 2.7. In Vitro Digestion of Oat Milk Tea Model System

#### 2.7.1. Simulated Gastrointestinal Tract Model

According to the in vitro assay methods described in earlier research [19,20], a gastrointestinal tract (GIT) model (oral, stomach, and small intestine) was used to simulate the digestion and absorption process of the oat milk tea model system in the gastrointestinal environment.

During the intestine phase, the pH was measured using an automatic titrator with magnetic stirring (Metrohm, CN Inc., Gladesville, Australia). The samples were maintained at a constant pH of 7.0 for 2 h by adding 0.25 N NaOH solution to the reaction vessel at 37 °C. The amount of free fatty acids (FFA) released during digestion was calculated based on previous studies [21].

#### 2.7.2. Microstructure

An optical microscope was used to observe the morphological structure of the samples at each stage of digestion. The objective was adjusted to 40× to observe the size and distribution of the droplets, and four different areas were selected for comparison for each sample and used to evaluate their properties.

#### 2.7.3. ζ-Potential

The surface potential (ζ-potential) of the mixed systems was measured using particle electrophoresis (ZSU5800, Malvern Instruments, UK). A buffer solution that was consistent with the pH of the digestion environment was used to disperse the samples from various stages, reducing multiple scattering effects.

#### 2.7.4. Bioaccessibility of Tea Polyphenols

The bioaccessibility of tea polyphenols after the in vitro digestion was determined by referring to a previous study [22]. A part of sample after the small intestine digestion was centrifuged (12,000 rpm/min, 20 min) to obtain the supernatant. The supernatant was considered as the mixed micelle fraction. Then, samples from the small intestine digestion and the supernatant were taken to determine the amount of tea polyphenols. The amount of tea polyphenols from the small intestine digestion and the supernatant were expressed as *C_Digesta_* and *C_Micelle_*, respectively. The bioaccessibility of tea polyphenols was calculated by Equation (2):

(2) $$\mathrm{Bioaccessibility}\;\left(\%\right)=\frac{C_{Micelle}}{C_{Digesta}}\times 100$$

### 2.8. Statistical Analysis

Experiments were repeated three times. Statistical analysis software (SPSS 25.0) with Tukey’s test was used for ANOVA (*p* < 0.05), and the experimental results were expressed as means and standard deviations.

## 3. Results

### 3.1. Binding of Tea Polyphenols with Oat Milk

**Figure 1:**
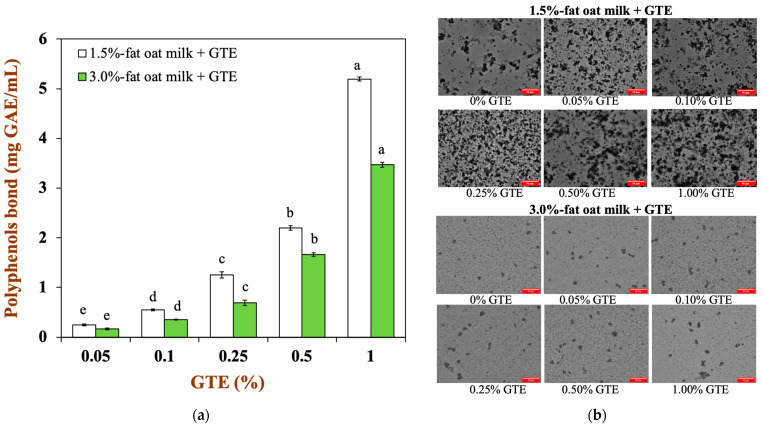
(**a**) The binding capacity of tea polyphenols with the two types of oat milk: (**b**) the microstructure of oat milk tea model system with two fat levels (scale label = 50 μm). Samples with different lowercase letters indicate significant differences (*p* < 0.05) when compared to various concentrations of green tea extract.

According to the previously described method, oat milk with different fat levels (1.5% and 3%) was mixed with a green tea extract (GTE) solution at a volume ratio of 8:2 (*v*/*v*), so that the GTE concentrations in the oat milk tea model system were 0.05%, 0.1%, 0.25%, 0.5%, and 1.0%. The binding of tea polyphenols with oat milk and the microstructure of the oat milk tea model system are shown in Figure 1. It can be clearly seen that the oat milk and tea polyphenols effectively bind (Figure 1a), and the quantity of bound tea polyphenols showed an upward trend as the GTE concentration increased in a given concentration range. Moreover, the range of polyphenols bound in the 1.5% fat oat milk tea model system was around 0.25–5.20 mg GAE/mL, while the range was approximately 0.17–3.50 mg GAE/mL in 3.0% fat oat milk tea model system. Therefore, 1.5% fat oat milk more easily bound with tea polyphenols than the 3.0% fat oat milk in the oat milk tea model system. As shown in Figure 1b, there were several large particles in 1.5% fat oat milk, and both the number and size of particles increased with the increasing GTE concentration. Compared with 1.5% fat oat milk, the particle size of 3.0% fat oat milk was smaller. Although the number of 3.0% fat oat milk particles rose slightly when mixed with GTE, the differences were not as noticeable as those noted when 1.5% fat oat milk was mixed with GTE. These results indicated that the fat content might be influenced the binding quantity of polyphenols and the aggregation of droplets in the oat milk model system. This phenomenon could be explained by the following reason: the hydrophobic region of the protein was absorbed to the lipid surface in the high fat food matrix [23], which weakened the hydrophobic interactions between proteins and polyphenols [24] and reduced the aggregation of the food matrix (Figure 1b).

### 3.2. Stability of Oat Milk Tea Model System

The optical centrifugation analysis technique uses a full-function stability analyzer, which can measure the instability of samples by centrifugal sedimentation. When infrared light passes through the sample tube, software can track changes in the light transmittance of the sample’s particles as they occur during centrifugation and plot those changes as a spectrum [25]. Infrared transmittance is the vertical coordinate, while the scale of the sample tube is represented by the abscissa. Each light line is scanned from the top to the bottom of the sample tube. The first line, which appears at the bottom of the image, is red, and the last line, which appears at the top of the image, is green. The entire spectrum depicts the path taken by the particles in the sample [26]. In addition, the particle movement rates reflect the relative stability; the lower the stability of the samples, the more the transmittance rate of sample changes during centrifugal acceleration [18].

**Figure 2:**
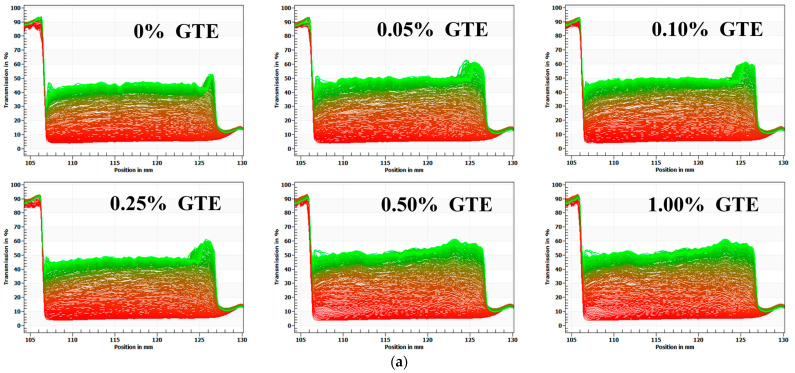

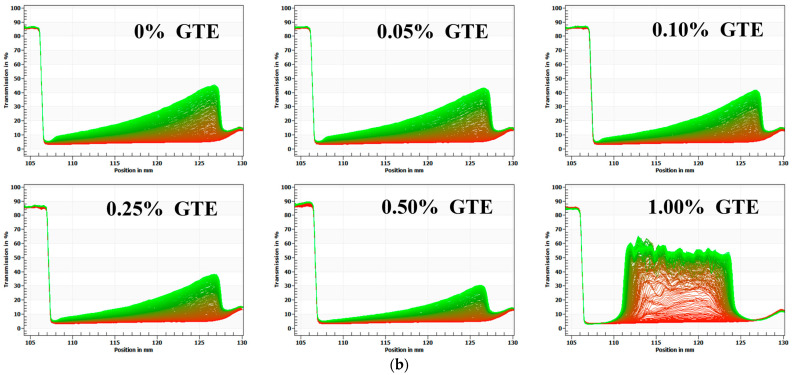
The physical stability of oat milk tea model systems: (**a**) 1.5% fat oat milk tea model system and (**b**) 3.0% fat oat milk tea model system.

As illustrated in Figure 2a, the transmittance rate of samples gradually increases as the centrifugation time goes on. The transmittance rate at the bottom of the test tube was significantly lower than the rest of test tube, indicating that a sediment layer was formed. When 1.5% fat oat milk was mixed with various GTE concentrations, the transmittance rate at the middle of the test tube slightly increased with increasing GTE concentration. The 1.5% fat oat milk tea model system was less stable than that of 3% fat oat milk, which was consistent with the results of the microstructure of the 1.5% fat oat milk tea model system. The presence of polyphenols in the emulsion with protein/polysaccharide induced the aggregation of particles, which resulted in the sample becoming unstable and phase separation occurring [27].

The transmittance rate of the sample changed dramatically with increasing GTE concentrations when 3.0% fat oat milk was mixed with various GTE concentrations (Figure 2b). Throughout the centrifugation procedure, the samples showed various degrees of phase separation. The infrared transmittance rate of the samples varied the most as the concentration of GTE reached 1.00%, suggesting that the centrifugal stability of the sample reduced [18]. Meanwhile, the LUMifuge analysis graphics of the 3% fat oat milk tea model system with 1.00% GTE showed a typical phase separation, with creaming at the top, water in the middle, and sediment at the bottom [28]. These results indicated that the physical stability of the 3.0% fat oat milk tea model system was almost completely destroyed when the GTE concentration reached 1.00%. Notably, the samples exhibited the best centrifugal stability when the GTE concentration was 0.5%,which indicated that the particles within the system had a better dispersion stability under centrifugal action.

As a result of the analysis above, it can be inferred that the 3.0% fat oat milk was more suitable for mixing with GTE to make a stable oat milk tea than the 1.5% fat oat milk. Moreover, excessive GTE addition (GTE concentrations > 0.50%) was not conducive to the stability of the oat milk tea model system.

### 3.3. In Vitro Gastrointestinal Digestion of Oat Milk Tea Model System

#### 3.3.1. Microstructure

**Figure 3:**
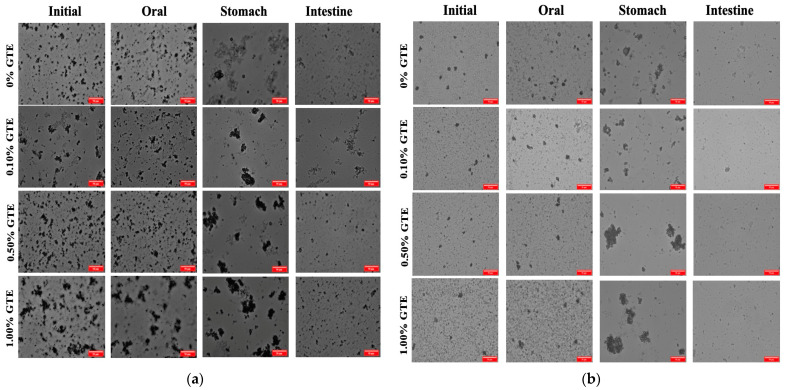
The optical microscope images of oat milk tea model systems after exposure to different in vitro digestive stages (scale label = 50 μm): (**a**) 1.5% fat oat milk tea model system and (**b**) 3.0% fat oat milk tea model system.

The microstructure of the oat milk tea model system at each stage of digestion is shown in Figure 3. At various stages of in vitro gastrointestinal digestion, the microstructure changes of the 1.5% fat oat milk tea model system (Figure 3a) and the 3.0% fat oat milk tea model system (Figure 3b) were comparable. The microstructures of the samples after exposure to the simulated oral environment did not alter significantly from the initial samples, indicating that the oat milk tea model system was tolerant to simulated oral conditions. Next, after exposure to the simulated gastric environment, the microstructures of samples were significantly different to those in the oral phase. There was an obvious increase in the droplets in each sample, which is a result of the aggregation of the proteins and oil droplets in the highly acidic conditions in the stomach. The causes of this phenomenon may be related to a lower electrostatic repulsion between droplets and other particles, or the pepsin that is present in the gastric juice breaking down proteins [19].

When the oat milk tea model systems transitioned from the stomach to the intestine environment, the microstructures and particle characteristics underwent further changes. After digestion by the small intestine, the pH increased to 7, which redispersed the droplets in the mixed systems. The microstructures showed that some of the droplets were not completely digested, which may be indigestible plant tissue fragments originating from the oat milk. The concentration of plant tissue fragments was significantly lower than that of the other colloidal components present (e.g., oil droplets), so that the initial samples may not have been detected. The dietary fibers that make up these bits of plant tissue are resistant to being broken down by amylases, proteases, and pancreatin in the gastrointestinal tract (GIT) [29]. In addition, there are also probably colloidal substances in the digesta, including digestive enzymes, calcium soaps, undigested lipid droplets, micelles, vesicles, liquid crystals, and other insoluble matter [30].

#### 3.3.2. ζ-Potential

**Figure 4:**
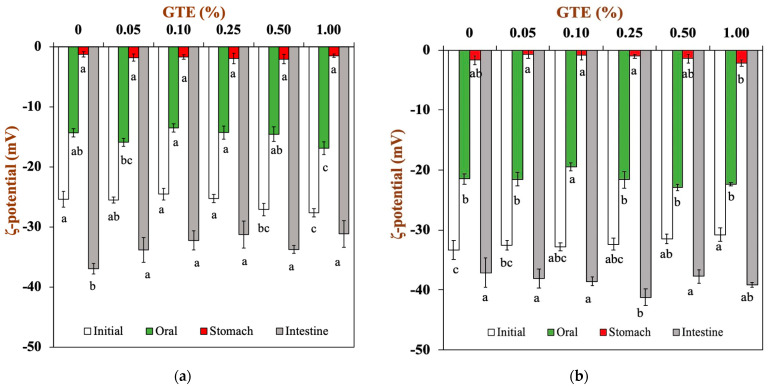
The ζ-potential of oat milk tea model systems after exposure to different in vitro digestive stages: (**a**) 1.5% fat oat milk tea model system and (**b**) 3.0% fat oat milk tea model system. Samples with different lowercase letters indicate significant differences (*p* < 0.05) when compared with various concentrations of green tea extract.

In order to provide indirect information regarding changes in the interfacial composition, the surface potentials of the oat milk tea model systems were measured as they progressed through the three stages of in vitro digestion. In the initial oat milk tea model system (pH 6.5-6.8), the droplets’ surface potential was dependent on their own characteristics (Figure 4). The ζ-potentials of the 1.5% fat oat milk tea model system were around −25 mV, whereas the ζ-potentials of the 3.0% fat oat milk tea model system were around −32 mV.

When the oat milk tea model systems were placed in a simulated oral environment (pH 6.8), all the droplets had a relatively low negative charge of around −15 mV for 1.5% fat oat milk tea model systems (Figure 4a) and around −20 mV for 3.0% fat oat milk tea model systems (Figure 4b). The observed changes in electrical charge on the droplets may be due to the changes in solution conditions (e.g., pH and ionic strength). The simulated saliva contains a variety of components that could potentially adsorb to droplet surfaces, including mucin. In fact, some studies have indicated that mucin can absorb to the surfaces of the droplets in emulsions through non-covalent interactions [31]. The reduction in the negative charge of the oat milk tea model system after digestion may be due to the interaction between the negatively charged mucin and the droplets of the oat milk tea model systems, where the mucin covers the interfacial layer of the oat milk tea model system and an electrostatic shielding effect occurs [32].

The electrical charge significantly lessened when the oat milk tea model system was transferred from the simulated oral environment into the gastric environment. The ζ-potentials of all the samples were around −0.7-−2.0 mV in the gastric environment. A previous study reported that oat-protein-coated droplets were positively charged at a pH of 3.0 (this pH value is similar to that of gastric fluid) because the pH is far below their isoelectric point (about 4.5) [33]. Therefore, the fact that the droplets in the oat milk tea model systems had a low negative charge indicated that either all or part of the protein had been removed from their surfaces or that the droplets had acquired a negatively charged coating (e.g., mucin) [34]. Additionally, the extremely low surface potential of the samples made the electrostatic repulsion between the droplets weaker, which resulted in the aggregation of droplets (Figure 3).

Next, the samples were transferred from simulated gastric fluids into simulated small intestine fluids. The pH value of samples returned to neutral (pH 7.0). For the 1.5% fat oat milk tea model system, the samples with GTE had a lower negative charge than the sample without GTE (Figure 4a). For the 3.0% fat oat milk tea model system, there was no obvious trend in the change in ζ-potential (Figure 4b). In general, all the samples had a relatively high negative charge in the simulated small intestine environment. The ionization of anionic groups (e.g., undigested protein, free fatty acids, bile salts, polysaccharides, and digestive enzymes) in samples digested in the small intestine was the primary cause of samples having a relatively high negative charge [22].

#### 3.3.3. Total Polyphenol Content and Polyphenol Bioaccessibility

**Figure 5:**
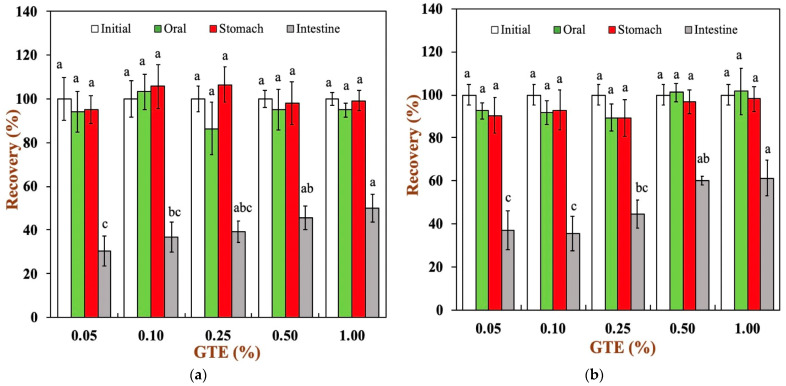

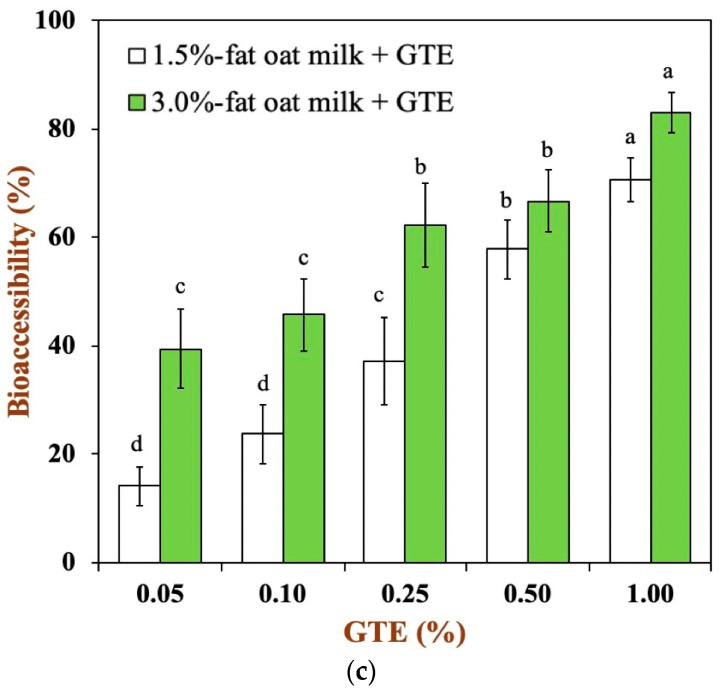
Total polyphenols recovery in oat milk tea model system after exposure to different in vitro digestive stages: (**a**) 1.5% fat oat milk tea model system and (**b**) 3.0% fat oat milk tea model system. (**c**) The bioaccessibility of tea polyphenols in two oat milk tea model systems. Samples with different lowercase letters indicate significant differences (*p* < 0.05) when compared to various concentrations of green tea extract.

The variation in total polyphenol recovery in two oat milk tea model systems at each digestive stage is presented in Figure 5a,b. As it can be observed, the polyphenol recovery was close to 100% in the simulated oral stage, characterized by simulated mastication and primary hydrolysis of glucose by α-amylase, which was mainly due to the short duration of this stage. In the gastric digestive stage, the well-known high stability of polyphenols against degradation under acidic conditions maintained a relatively high polyphenol recovery [35]. Finally, there was significant decline in the polyphenol recovery during digestion in the small intestines. For all samples, the polyphenol recovery ranged from 30.61 to 61.25% at the end of small intestinal digestion. The main reason for this phenomenon is the degradation of polyphenols at 2 h of small intestinal digestion. The neutral pH, the residual dissolved oxygen, and the reactive oxygen accelerated the degradation of polyphenol in the small intestinal fluid by auto-oxidation and epimerization reactions [9,36]. The polyphenol recovery was only 30.61% for the 1.5% fat oat milk tea model system with 0.05% GTE and 36.80% for the 3.0% fat oat milk tea model system with 0.05% GTE. There was lower polyphenol recovery in oat milk tea model systems with low GTE concentrations in comparison to those with high GTE concentrations. It is worth noting that the 3.0% fat oat milk tea model system with 0.50% and 1.00% GTE presented the highest total polyphenol recovery. The reason for this result may be related to the initial concentration of GTE.

To further understand the efficiency of the oat milk tea model system in digestion, the bioaccessibility of green tea polyphenols was determined at the end of intestinal digestion, as shown in Figure 5c. In the oat milk tea model systems with different fat contents, the bioaccessibility of green tea polyphenols increased with an increase in the polyphenol concentration. The bioaccessibility of tea polyphenols in the 1.5% fat oat milk tea model system ranged from approximately 14.18 to 70.53%, while the bioaccessibility of tea polyphenols was about 39.45–83.03% for all samples in the 3.0% fat oat milk tea model system. It can be clearly seen that the 3.0% fat oat milk tea model system has a higher bioaccessibility of tea polyphenols. It is speculated that this may be related to the interaction between food matrices and polyphenols. A variety of food matrices, such as dietary fiber, large peptide residues, and fatty acids, that absorb polyphenols inside the gastrointestinal tract have a great impact on the bioaccessibility of polyphenols [9,37]. In our study, compared with the 1.5% fat oat milk tea model system, the 3.0% fat oat milk tea model system has a lower binding capacity with tea polyphenol (Figure 1a) and a higher polyphenol recovery during small intestine digestion (Figure 5a,b). As the result of these two factors, the bioaccessibility of tea polyphenol in the 3.0% fat oat milk model system was higher than that of the 1.5% fat oat milk model system, especially in 3.0% fat oat milk model systems with 0.05–0.25% GTE. Similar results were observed in the study of Ortega, N. et al., who found that a higher fat content in cocoa liquor seemed to lead to a better bioaccessibility of cocoa polyphenols [38].

#### 3.3.4. Release of FFA

**Figure 6:**
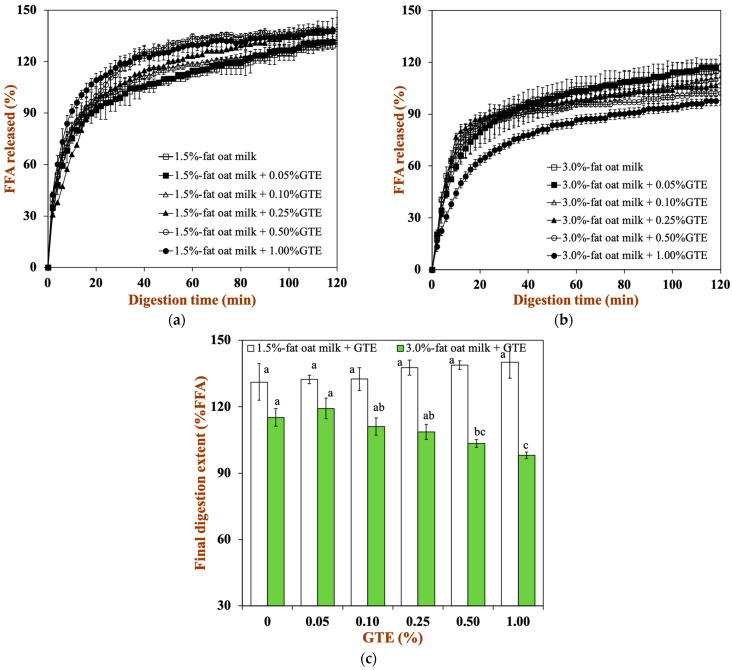
The total amount of FFA released from two oat milk tea model systems with various GTE concentrations: (**a**) 1.5% fat oat milk tea model system and (**b**) 3.0% fat oat milk tea model system. (**c**) Final extent of FFA release after exposure to 2 h of small intestine digestion. Samples with different lowercase letters indicate significant differences (*p* < 0.05) when compared to various concentrations of green tea extract.

A pH-stat method was used to track the FFA release profile to investigate the impact of dairy matrices and GTE concentration on lipid digestion (Figure 6a,b). All samples showed similar digestion profiles, from which two phases could be clearly distinguished. The first phase of the digestion profile is related to a high lipolysis rate due to a fast adsorption of pancreatin onto the droplets surface and a rapid release of FFA into the continuous phase. Afterwards, the lipolysis rate tends to constant values because the products generated during the digestion (e.g., FFA) limit pancreatin adsorption and reduce enzyme activity [39].

To a certain extent, different food matrices and GTE concentrations also affect the rate of lipid digestion. With higher fat levels in oat milk, the final extent of lipid digestion clearly dropped: for 1.5% fat oat milk, it was about 131.15%, and for 3.0% fat oat milk, it was about 115.17% (Figure 6c). This might be due to the reduced ratio of lipase to lipid in 3.0% fat oat milk, resulting in a low extent of lipid digestion [21]. Moreover, the final extent of lipid digestion did not vary significantly in the 1.5% fat oat milk tea model system. Conversely, the final extent of lipid digestion for the the 3.0% fat oat milk tea model system fell from 119.25% in the sample with 0.05% GTE to 98.03% in the sample with 1.00% GTE. This result suggested that the addition of GTE remarkably reduced the FFA release from the oat milk tea model system, especially in the 3.0% fat oat milk tea model system. Among the four major polyphenols (epigallocatechin-3-gallate: EGCG, epigallocatechin: EGC, epicatechin-3-gallate: ECG, and epicatechin: EC) in green tea, pancreatic lipase activity has been reported to be inhibited by EGCG and EGC, which is due to the fact that tea polyphenols may bind to the enzymes close to the active site and reduce their activity [40]. Additionally, tea polyphenols also interfere with the emulsification and micellar solubilization of lipids, which influences fat digestion and absorption processes [41]. Therefore, the lower ratio of lipase to lipid and the inhibitory effect of polyphenols on lipase activity in the 3.0% fat oat milk tea model system led to the a clearer influence of tea polyphenols on lipid digestion compared to the 1.5% fat oat milk tea model system.

## 4. Conclusions

The results of our study revealed that the stability, lipid digestion, and tea polyphenol bioaccessibility of oat milk tea model systems depended on oat milk matrices and the concentration of green tea extract (GTE). In terms of the oat milk, 3.0% fat oat milk was more appropriate for use in the formulation of oat milk tea than 1.5% fat oat milk, because it showed better stability and bioaccessibility of tea polyphenols after mixing with GTE concentrations of <0.50%. However, a high concentration of GTE (GTE ≥ 0.50%) reduced the final extent of lipid digestion, especially in 3.0% fat oat milk. Therefore, selecting suitable oat milk matrices and GTE concentrations can maintain the stability of oat milk tea model systems and improve the bioaccessibility of tea polyphenols in oat milk tea model systems. The aforementioned findings may be crucial when designing plant-based milk tea for consumers who are lactose intolerant, vegan, or allergic to bovine milk proteins.

## Figures and Tables

**Table 1:** Nutrition information per 100 mL of oat milk.

Name	Protein	Fat	Carbohydrate	Dietary Fiber	Sodium	Calcium
1.5% fat oat milk	1.0 g	1.5 g	6.6 g	0.8 g	42 mg	120 mg
3.0% fat oat milk	1.0 g	3.0 g	6.5 g	0.8 g	42 mg	120 mg

## Data Availability

The data are contained within the article.

## Abbreviations

GTE, green tea extract; TPs, tea polyphenols; FFA, free fatty acids; GAE, gallic acid equivalent.
